# Priming of CD8^+^ T Cell Responses to Liver Stage Malaria Parasite Antigens

**DOI:** 10.3389/fimmu.2014.00527

**Published:** 2014-11-05

**Authors:** Giampietro Corradin, Jelena Levitskaya

**Affiliations:** ^1^Biochemistry Department, University of Lausanne, Epalinges, Switzerland; ^2^W. Harry Feinstone Department of Molecular Microbiology and Immunology, Bloomberg School of Public Health, Johns Hopkins University, Baltimore, MD, USA

**Keywords:** *Plasmodium*, malaria, liver stage antigens, antigen presentation to T cells, dendritic and liver cells as APC

## Abstract

While the role of malaria parasite-specific memory CD8^+^ T cells in the control of exo-erythrocytic stages of malaria infection is well documented and generally accepted, a debate is still ongoing regarding both the identity of the anatomic site where the activation of naive pathogen-specific T cells is taking place and contribution of different antigen-presenting cells (APCs) into this process. Whereas some studies infer a role of professional APCs present in the lymph nodes draining the site of parasite injection by the mosquito, others argue in favor of the liver as a primary organ and hepatocytes as stimulators of naïve parasite-specific T cell responses. This review aims to critically analyze the current knowledge and outline new lines of research necessary to understand the induction of protective cellular immunity against the malaria parasite.

## Introduction

It is currently accepted that priming of CD8^+^ T lymphocytes by antigen-presenting cells (APCs) takes place in the secondary lymphoid organs such as spleen and lymph nodes [reviewed in Ref. (1)]. Multi-photon-based intravital microscopy revealed that the first contact between naïve CD8^+^ T cells and APC takes place in the periphery of draining lymph nodes (DLN) shortly after infection and mainly occurs in the subcapsular sinus or the interfollicular regions enriched with pathogen-derived antigens (2, 3). Depending on the pathogen’s nature, the rapid relocation of naïve T cells to the periphery of the draining lymph node can be either antigen-specific (2) or antigen-independent, associated with decreased local levels of chemokines and the drastic alteration of the lymph node architecture by the pathogen (3, 4). Data from mice infected with vesicular stomatitis virus demonstrated that, though CD169+ macrophages (5) residing in the subcapsular sinus were the major cell population bearing virus-derived antigens (2), dendritic cells (DCs) served as the primary APC triggering antigen-specific naïve CD8^+^ T cells. The ability of immature dendritic cells to acquire exogenous antigens followed by their proteolytic processing and presentation on the MHC class I molecules, commonly referred to as “cross-presentation,” is believed to be the major requirement for the generation of primary antigen-specific CD8^+^ T cell responses against pathogens (6–9).

Upon the initial encounter of naïve T cells with APC, a heterogeneous progeny of antigen-specific CD8^+^ T cells including short-lived effector cells (SLEC) and memory precursor effector cells (MPEC) [reviewed in Ref. (10, 11)] is generated. It is still not clear whether the SLEC versus MPEC differentiation is enforced by the asymmetric segregation of transcription factors and protein degradation machinery already at the first cell division (12–14) or it reflects the differential exposure to inflammatory and co-stimulatory “help” signals received from APCs by antigen-specific CD8^+^ T cells during the expansion phase [reviewed in Ref. (10, 11)]. While generation of primary CD8^+^ T cell responses to non-inflammatory antigens requires CD4^+^ T cell help, induction of primary CD8^+^ T cells responses to *Listeria*, LCMV, and influenza virus is CD4^+^ T cell-independent and results from direct activation of APCs by the pathogen (15–17). Moreover, CD4^+^ T cell help can be replaced by the CD40 triggering on the DCs, which prime antigen-specific naïve CD8^+^ T cells (18, 19). Thus, the exact nature and requirements for “help” signals necessary for the initial triggering and subsequent expansion of primary antigen-specific CD8^+^ T cell responses vary among different pathogens and sites of primary infection. In this report, our objective is to present and discuss the published data regarding CD8^+^ T cell activation in *Plasmodium* infection, and suggest experiments to better understand the antigen presentation process.

Malaria infection is initiated through the bites by *Plasmodium*-carrying female Anopheles searching for blood to support egg development. As the mosquito probes the host environment under the skin for the presence of blood vessels, it injects salivary gland proteins both prior and during blood feeding to inhibit blood coagulation. Parasites deposited into the skin can also traverse surrounding cells and enter the circulation with subsequent infection of liver cells. Studies performed with parasites injected intradermally or intravenously show that the resulting liver parasite load is similar (20). In addition, transfer of parasites from the skin sites to DLN occurs (21).

Identification of the anatomical site and the type of APC, which orchestrate the induction of primary CD8^+^ T cell responses against a particular antigen, represents an essential step in rational design of CD8^+^ T cell-based vaccination strategies. Whereas the research on the effector phase of CD8^+^ T cell response against malaria has been quite extensive (22–25), a rather limited number of studies attempted to dissect the issue of liver stage-specific CD8^+^ T cell priming in the infected host.

## Role of Different Organs in Antigen Presentation

In this respect, the study by Chakravarty et al. (21) appears to be one of the most comprehensive and systematic up to date. The authors concluded that extrahepatic lymphoid tissues, in particular the DLN and spleen are the most important sites contributing to the generation of the effector T-cell pool in the liver. In agreement with these data, Obeid and colleagues demonstrated that strictly subcutaneous immunization with irradiated sporozoites led to induction of sterile immunity against pre-erythrocytic malaria with T cell priming occurring in skin-draining lymph node (26). It was proposed that parasite-specific CD8^+^ T cell priming depends on cross-presentation of malaria antigens (21). This indicates that professional APC, rather than infected hepatocytes, trigger priming of naïve CD8^+^ T cells directed to liver stage antigens.

Several lines of experimental evidence were presented in support of these conclusions. Thus, IFNγ production by adoptively transferred circumsporozoite protein (CSP)-specific naïve transgenic T cells was first detected in the skin-DLN as early as on day 2 after mouse immunization by microinjection or mosquito bites, whereas no detectable T cell activation was detected in other organs including spleen. Hence, Chakravarty and co-authors suggested that these temporal differences in the onset of parasite-specific T cell activation could reflect the hierarchical order of T cell priming initiated in the DLN that could be followed by migration of primed CD8^+^ T cells to other organs, including the spleen and the liver. However, removal of lymph nodes draining the site of parasite injection prior to the adoptive transfer of parasite-specific CD8^+^ T cells, though resulted in a 60% reduction of activated CD8^+^ T cells in the liver, did not affect the frequencies of primed CD8^+^ T cells in the spleen where the first signs of T cells activation were documented only 24 h later than in DLN and at the same time point as in the liver. These data indicate that temporal differences in the onset of T cell activation used as a parameter for identification of the CD8^+^ T cell priming site should be carefully reconsidered in future studies and further strengthen the importance of the spleen as a site of induction of primary CD8^+^ T cell responses in animal models of the infection. The latter is in agreement with the data by Sano et al. (27) demonstrating that spleens of infected mice support priming of parasite-specific naïve CD8^+^ T cells following intravenous injection of sporozoites.

At the same time, several lines of evidence presented by Chakravarty and colleagues (21) do not firmly support the essential role of the spleen in the parasite-specific CD8^+^ T cell priming.

First, DCs isolated from the spleens 60 h after injection of sporozoites were unable to trigger proliferation of parasite-specific CD8^+^ T cells, whereas DCs isolated from the DLN efficiently induced T cell proliferation and, presumably, presented the antigen. Since no data with liver-resident DCs were generated, a direct role of intrahepatic professional APCs in priming of parasite-specific CD8^+^ T cells still needs to be addressed. In addition, as the first signs of activation of parasite-specific T cells in DLN were detected at day 2 post immunization, it is not completely clear whether DCs from DLN had a greater capacity to prime CD8^+^ T cells as compared to spleen and liver-resident DCs at time points earlier than 60 h.

Second, animals subjected to simultaneous lymphadenectomy and splenectomy prior to the adoptive transfer of CSP-specific CD8^+^ T cells followed by immunization with sporozoites and subsequent challenge with viable parasites 10 days later had similar load of parasites in the liver as non-immunized mice, indicating that either DLN or/and spleen are required for CSP-specific CD8^+^ T cell priming. At the same time, splenectomy alone did not affect inhibition of parasite development in the liver, prompting the authors to conclude, that DLNs are the priming site of protective CD8^+^ T cell responses.

Interestingly, as shown by Chakravarty and co-authors (21), removal of both DLNs and the spleen prior to immunization with sporozoites, though drastically reduced the activated T cell pool in the liver, failed to abrogate it completely, suggesting that at least a proportion of parasite-specific CD8^+^ T cells found in the liver had been primed outside the DLN and the spleen. These findings could reflect the process of parasite-specific CD8^+^ T cell triggering in the liver and define it as the organ essential for the parasite development. On the other hand, animals treated with FTY720, a drug, which inhibits lymphocyte egress from lymph nodes (28, 29), had substantially less IFN gamma producing parasite-specific CD8^+^ T cells at day 7 post injection with irradiated sporozoites. Based on this observation, the authors concluded that systemic distribution of CD8^+^ T cells, at least in part, contributes to the intrahepatic pool of parasite-specific CD8^+^ T cells (21). It still needs to be seen, if treatment with FTY720 (30) inhibits the development of “early-primed” parasite-specific CD8^+^ T cells in the liver and spleen, previously noted by the authors already 72 h after mosquito bite. In addition, effect of FTY720 on the protection of animals from subsequent challenge with live sporozoites has to be addressed in this model. Noteworthy, the time course of the parasite-specific clonal T cell activation in the lymph nodes, liver, and other organs is only slightly delayed (by 24 h) while it is known that activated T cells egress from the lymph nodes 4–5 days after antigen encounter (31, 32). The latter suggests that either activation of parasite-specific T cells may take place simultaneously in various organs, or unusually rapid egress from the lymph node after priming is an intrinsic feature of T cells in this specific experimental model.

## Role of Infected Hepatocytes in Antigen Presentation

The role of infected hepatocytes in direct priming of naïve parasite-specific CD8^+^ T cells is still a subject of controversy. Early study by Renia et al. demonstrated that intrasplenic injection of infected hepatocytes induced protective T cell-mediated immunity against infection with *Plasmodium yoelii* and *P. berghei* sporozoites (33). Leiriao et al. demonstrated that apoptotic hepatocytes infected with irradiated sporozoites are phagocytosed by DCs and merely serve as a source of *Plasmodium* antigens for the initiation of the protective immune responses via cross-priming (34). In contrast, Renia and collaborators argued against apoptotic infected hepatocytes as a source of antigens and suggested that liver DCs could be activated upon uptake of parasite antigens directly from viable infected hepatocytes (35) as previously seen in other experimental models (36, 37). However, data from Chakravarty et al. implied that though cross-priming is required, it takes place in the DLNs and not in the liver (21). In agreement with these data, Jung et al. demonstrated that mice subjected to chemical depletion of CD11c^+^ DCs fail to induce CD8^+^ T cell responses to infection with *Plasmodium yoelii* (38). Neither of these studies considered hepatocytes as an APC subset capable of initiating the primary parasite-specific T cell responses.

A recent study by Balam et al. (39) focused on two questions: can infected hepatocytes directly prime naïve parasite-specific T cells and does stimulation of already primed CD8^+^ T cells protect mice against parasite challenge? Administration of CD8^+^ CSP-specific T cells but not an irrelevant T cell clone injected into TAP-deficient MHC class I mismatched recipient mice, simultaneously with infected hepatocytes bearing MHC haplotype relevant for parasite-specific T cells, resulted in 100% protection of mice from subsequent challenge with live sporozoites (39). As the observed protection was not due to a bystander effect or a continuous cytokine secretion by parasite-specific CD8^+^ T cells, these data demonstrate that infected hepatocytes are capable of presenting the antigen to CD8^+^ T cells, reactivating resting CSP-specific CD8^+^ T cells and inducing protection.

Importantly, more than 60% of naïve BALB/c mice injected with irradiated sporozoite-infected hepatocytes were also protected from subsequent live parasite challenge, suggesting that infected hepatocytes could contribute to the priming of endogenous naïve T cell. However, T cell depletion experiments are required to confirm that protection is T cell-mediated. Finally, to formally exclude contamination with other APC potentially present in the hepatocyte preparations and capable of presenting CSP and priming the naïve CD8^+^ T cells, isolation of pure hepatocyte population devoid of cells bearing markers of DCs, macrophages, and stellate cells should be done by flow cytometry using fluorescent transgenic parasites. On the other hand, arguing against the sole role of professional APC in priming of naïve immune responses to malaria parasites, mice depleted of DCs by treatment with cytochrome *c* were still protected from the challenge with live sporozoites in spite of significantly lower frequencies of endogenous parasite-specific T cells primed by the immunization with irradiated sporozoites (39). These data do not fully support the previously discussed role of dendritic cell function in induction of primary malaria liver stage-specific T cell responses (21, 38).

## Other Considerations

The quality of hepatocytes as APCs capable of triggering T cells responses had been recently dissected by Ma et al. (40). It had been demonstrated that *P. berghei* and *P. falciparum* infected human hepatocytes retain largely unaltered expression of multiple molecules of the MHC class I pathway until very late stages of parasite development (40). Moreover, infected cells exhibited no obvious defects in the capacity to upregulate expression of different molecular components of the MHC class I machinery in response to pro-inflammatory lymphokines or trigger direct activation of allo-specific as well as peptide-specific human CD8^+^ T cells (40). At the same time, it is not known whether or not the characteristic features of professional APC believed to be important for efficient T-cell priming, i.e., co-stimulatory molecules B7.1 and B7.2 (“signal 2”), as well as production of cytokines essential for the survival and maintenance of primed T cells (“signal 3”) are possessed by the primary human hepatocytes *in vivo* and/or induced upon infection.

Current literature dissecting the ability of primary hepatocytes to specifically prime naïve CD8^+^ T cells is scarce. Bertolino et al. demonstrated that purified primary murine hepatocytes were able to induce activation and proliferation of antigen-specific naive CD8^+^ T cells *in vitro*, even in the absence of exogenously added cytokines as well as CD80 and CD86 co-stimulatory molecules (41). Moreover, the magnitude of T cell proliferation induced by primary hepatocytes was comparable to that induced by DCs. Naïve T cell priming by hepatocytes did not require CD4^+^ T cell help and induced expression of early T cell activation markers and transient CD8^+^ T cell effector activity followed by rapid cell death of activated T cells. Thus, primary hepatocytes were able to prime naïve T cells but failed to sustain productive antigen-specific CD8^+^ T cell responses (41). In agreement with these data, *in vivo* experiments using endogenous expression of alloantigens under hepatocyte-specific promoters demonstrated that activation of primary T cells by hepatocytes as antigen-presenting cells leads to T cell apoptosis rather than formation of antigen-specific memory T cell pool (42–44). It was further demonstrated that T cells activated by hepatocytes died “by neglect” and lack of IL-2 and low expression of pro-survival genes due to insufficient co-stimulation during the priming phase (45). Hence, taking into account the inability of primary hepatocytes to provide appropriate co-stimulation during T cells priming along with the immunosuppressive microenvironment created by multiple subsets of the liver-resident APC [reviewed in Ref. (46, 47)], it may appear unlikely that hepatocytes infected with malaria parasites play a major role in the generation of effective parasite-specific CD8^+^ T cell memory responses. However, it does not preclude the possibility that CD8^+^ T cells specific to malaria antigens could be primed and activated, at least shortly, by hepatocytes supporting development of exo-erythrocytic forms. Indeed, given proper stimuli, such T cells can be rescued to full immunological competence and longer survival (48, 49). In the case of malaria, proper activation stimuli could be induced by *Plasmodium* infection leading to activation of numerous genes in hepatocytes (50, 51) including those involved in native immunity and antigen presentation. Since no transcriptional analysis has been performed in Kupffer cells traversed by sporozoites so far, it would be important to understand whether or not liver-resident macrophages change their immunomodulatory properties in the site of malaria infection.

At this point, a word of caution should be expressed to the fact that all animal studies discussed above were based on a single mouse strain, BALB/c, as well as a single CD8^+^ T cell epitope derived from the CSP. Future studies on the induction of primary T cell responses to exo-erythrocytic forms of malaria need to be extended to other protective CD8^+^ T cell epitopes including responses, which appear later in the liver stage by using either radiation attenuated (RAS) or genetically attenuated (GAS) sporozoites or sporozoites combined with chloroquine chemoprophylaxis (CPS) (52).

## Field Studies

It is still unclear to what extend animal models dissecting induction of primary T cell responses to malaria as well as human studies involving vaccinated volunteers reflect the acquisition of natural cellular immune responses in malaria endemic areas. An acquisition of a sterile immune protection following immunization with RAS, GAS, or using CPS regime in animals and humans sharply contrasts with the situation in the field, where, in spite of frequent (up to 30 per month in certain areas) biting by infected mosquitoes (53, 54), no sterile protection is usually obtained in both adults and children in response to natural infection under drug treatment or intermittent preventive treatment (IPT). Several hypotheses could be proposed to explain this discrepancy: (1) sporozoite “charge” (and, as a result, supply of parasite antigens) is too small in the field as compared to that given under experimental conditions; (2) down-regulation of the parasite-specific CD8^+^ T cell responses by the content of mosquito salivary glands delivered together with sporozoites to the site of T cell priming; and (3) excessive and/or preceding induction of immune responses to salivary gland proteins. As for the latter, given the fact that only a fraction (0–25% depending on the seasons and location) of mosquitoes are infected (54–56), a memory T-cell pool specific for salivary gland antigens is most likely established prior to parasite infection. As a result, secondary T cell responses directed to mosquito antigens could be preferentially activated at the expense of parasite-specific T-cell activation via, for example, competition for IL-2, homeostatic niche, or by active secretion of inhibitory molecules (57–64). If this is the case, the efficacy of sporozoite-based pre-erythrocytic vaccines may turn out to be low in endemic areas due to even subtle contamination with the salivary gland proteins. Both the second and third hypotheses may explain the low frequency of parasite-specific helper and CD8^+^ T cell found in humans from malaria endemic regions, as well as the general failure [with the exception for a single donor so far (65)] to obtain stable human T-cell clones specific to malaria liver stage antigens.

## Final Remarks

In conclusion, existing experimental data obtained from animal models suggest that: (1) both DCs and hepatocytes can prime naïve malaria parasite-specific CD8^+^ T cells, at least those directed to epitopes derived from CSP and (2) either DCs or hepatocytes are sufficient to induce protective CSP-specific T cell responses if the parasite load is not excessive. Identification of the essential site for priming of malaria liver stage-directed CD8^+^ T cell responses of broader antigen specificity as well as mimicking the conditions of the natural exposure to the uninfected mosquito vector will pave the way for the optimal design of T cell-based vaccines. We hope that experimental approaches suggested above in the context of the reviewed original data (21, 27, 34, 39) will prompt further studies on the induction and maintenance of protective T cell responses against exo-erythrocytic stages of malaria infection.

## Conflict of Interest Statement

The authors declare that the research was conducted in the absence of any commercial or financial relationships that could be construed as a potential conflict of interest.
